# Effect of Photo-Selective Shade Nets on Pollination Process and Nut Development of *Corylus avellana* L.

**DOI:** 10.3389/fpls.2020.602766

**Published:** 2020-12-10

**Authors:** Devid Guastella, Mbuyseli Sigwebela, Eloy Suarez, Oscar Stubbs, Jorge Acevedo, Gerhard Engelbrecht

**Affiliations:** ^1^Agrisudafrica (Pty) Ltd., Franklin, South Africa; ^2^Ferrero Hazelnuts Company, Division of Ferrero Trading Luxembourg, Senningerberg, Luxembourg

**Keywords:** hazelnut, pollen tube, abortion, ovule, aniline blue, disodium fluorescein, assisted pollination

## Abstract

Hazelnut (*Corylus avellana* L.) is one of the most appreciated nut crops, which is motivating the cultivation outside its historical production areas. Despite that, there is still limited knowledge about the floral biology of the species and its developmental fruiting stages under different environments. Adverse climatic conditions can threaten the pollination process and fruit development. In South Africa, the deciduous fruit industry identified the net shading as a tool to mitigate the effects of unfavorable abiotic events. The objective of this work was to investigate the effects of photo-selective nets on the pollination process and nut development of *C. avellana*. Mature hazelnut trees were maintained under netting and compared with the ones in open field. Microscopic examination of female flower and developing nuts were conducted in order to observe the pollen tube growth and the pattern of disodium fluorescein transport into the funiculus and ovule. The results showed differences in pollen tubes growth and timing between the treatments. Generally, trees under nets showed higher rate in pollen tubes developing and reaching the base of the style. On the contrary, the tests carried out in open field showed a higher ratio of pollen tubes arrested in the style. The results also indicated differences in ovules abortion. Developing fruits that showed an interruption point at the funicle level or at junction point of the ovule were classified as aborting fruits (blank nuts at harvest time). A higher rate of abortion was detected in open field compared to the plants under netting. In conclusion, the shade nets influenced the pollen tube growth and the nut development, principally due to micro-climate modification. Therefore, further investigations are needed to analyze the influence of light spectra and to determine the sustainability of photo-selective nets over several years.

## Introduction

The implications of global warming are becoming major issues on the productivity of temperate fruit industry, which may decline because of the sensitivity to higher temperatures, heat waves, frequent frost, hail and strong wind events (Luedeling et al., 2011; Hribar and Vidrih, 2015; Rai et al., 2015; Midgley et al., 2016). In the South Hemisphere, South Africa is one of the largest exporting countries of temperature fruits, second only to Chile (Retamales, 2011). Therefore, during last years, extreme climatic events such as El Niño, warmer winter conditions, drought, and hailstorms caused significant economic losses (Midgley et al., 2016). In addition, future projections are calling for effective contingency plans to be implemented in the fruit production systems (Gbetibouo and Hassan, 2005; Luedeling, 2012; Calzadilla et al., 2014; Mditshwa et al., 2019). For these reasons, the implementation of shade nets (antihail, insect screen, and photo-selective) is becoming popular in the fruit industry, for both temperate and sub-tropical fruit crops (Stander and Cronje, 2016; Tinyane et al., 2016; Brown, 2018; Mditshwa et al., 2019). The temperate tree nut industry is expanding worldwide. In South Africa, the predominant nut crops grown are macadamias (world’s largest exporter) and pecans (Brits, 2018; Sikuka, 2020). However, other nuts such as walnut, almond, pistachio, and hazelnut, relatively new in South Africa, are slowly emerging, where research institutes, private companies, and farmers are working on finding appropriate agronomic procedures, cultivars, and to demonstrate the sustainability of the crop (Swart and Blodgett, 1998; Chen and Swart, 2000; Ascari et al., 2018; Kriel, 2019). Although recent reports from the International Nut and Dried Fruit Council [INC] (2020) point out that traditional hazelnut growing countries like Turkey, Italy, Azerbaijan, and Georgia source 88% of the global production, the increasing interest toward hazelnut has driven the search for new areas of cultivation outside its native range. European Hazelnut (*Corylus avellana* L.) is a monoecious and wind pollinated species. Its floral biology exhibits several unusual characteristics (Germain, 1994). In the north hemisphere, the male inflorescences (catkins) induction starts in mid-May and they begin to be visible in June, reaching the maturity in winter. Consequently, pollination occurs during winter, as early as middle November in some cultivars in the Northern hemisphere (Cristofori et al., 2018) or in June for the Southern hemisphere (Von Bennewitz et al., 2019). Hazelnut floral biology is quite uncommon, where a considerable lapse of time occurs between pollination and fertilization. In fact, soon after the pollination, the pollen tube grows to the base of the style, where the tube becomes latent and waits for the ovary to be mature (Rigola et al., 2001; Tiyayon, 2008; Liu et al., 2014). Only then, the fertilization takes place. Therefore, this delayed fertilization is between the principal factors threatening hazelnut yield (Heslop-Harrison et al., 1986; Çetinbaş-Genç et al., 2019). Pollination and fertilization processes are extremely sensitive to air temperature and relative humidity (RH) (Kelley, 1979; Huo et al., 2014; Çetinbaş-Genç et al., 2019); for these reasons, specific attention should be given to protect the trees from abnormal and extreme climatic conditions. While there is extensive literature on the effects of shading nets on floral development of several fruit species (Mupambi et al., 2018; Manja and Aoun, 2019; Mditshwa et al., 2019), very few applied researches were carried under nets on hazelnuts (Hampson et al., 1996; Azarenko et al., 1997; Me et al., 2005). This study aims to explore the effect of protective netting on the reproductive development phases of hazelnut as mitigation technique against erratic weather conditions.

## Materials and Methods

### Site Description and Plant Material

The trials were carried out in a 9-year-old hazelnut orchard, at Agrisudafrica Ltd., an experimental farm situated in Greater Kokstad Municipality of KwaZulu-Natal Province, South Africa (30°21′34.63”S; 29°25′31.22”E), altitude 1560 m above sea level. According to Köppen-Geiger climate classification, updated by Kottek et al. (2006) and Rubel et al. (2017), the local climate is classified as *Cw* (Warm temperate climate with dry winter). Twenty hectares of orchard were covered with photo-selective nets and 10 ha in open field were considered as control. Since the performance of protective nets and their influence on certain physiological parameters of a tree seem to vary based on the geographical location (Meena et al., 2015; Zoratti et al., 2015; Brkljača et al., 2016), and in the absence of any existing local information, it was decided to verify the effects of different photo-selective nets. Each block had a size of 2 ha. The experiment started in June 2018 and ended in January 2020. The trials were set considering two hazelnut cultivars: Tonda di Giffoni and Barcelona (also known as Fertile De Coutard). The tree spacing between and within the rows was 5 × 3 m for Tonda di Giffoni (665 trees/ha) and 5 × 4 m for Barcelona (500 trees/ha). In the orchard, four blocks of Tonda di Giffoni and four blocks of Barcelona cultivar were selected for the experiment. Each block was divided in three sections, one for each treatment: Active Blue (20%), Photo Red (20%), and Black/White 20%, from Knittex (South Africa). Each tree row in a section included 66 trees for Tonda di Giffoni and 50 for Barcelona. Each net was covering at least five rows. The brand name “SpectraNets” describes the capacity of the nets to manipulate the quantity, quality, and relationship of blue, green, red, and far-red wavelengths (Knittex, 2020). The characteristics of each net are described in Table 1. The effect of the nets was compared with trees grown under sunlight. For each treatment, temperature and humidity in open field and under the nets were monitored hourly using data loggers (Tinytag Plus 2, Gemini Data Loggers Ltd., United Kingdom). Data loggers were installed 2 m above the ground, at the center of each netted section. In addition, Arable Mark 2 weather stations (Arable Labs, Inc., San Francisco, CA, United States) was installed in the orchard in open field. The soil texture was clay with a pH ranging from 5 to 5.6 between the blocks and electrical conductivity (EC) of ±0.3 mS/cm. All trees in the orchard were fertigated with the same nutritional solution. The irrigation timing and fertigation were managed by a drip irrigation system and controlled automatically using Irricheck Pulse^TM^, a platform that allows to use different combinations of soil moisture probes, sensors, and telemetry, helping to maintain soil moisture at optimal levels. Soil and irrigation management information for the dry winter season are summarized in Table 2.

**TABLE 1:** Net details.

Net	Shade factor %	Blue wavelength transmittance % (450–495 nm)	Red wavelength transmittance % (620–760 nm)	UV block
Active Blue	20	90	83	20
Photo Red	20	79	84	23
Black/White	20	77	78	27

**TABLE 2:** Irrigation water management during dry winter season.

Soil texture	Soil water thresholds*			m^3^/block/day		m^3^/block/season		Rainfall
			
	*FC%*	*PWP%*	*TAW%*	Control	Netted area	Control	Netted area	(mm)
Clay	45	25	20	35	20	4305	2460	58.8
Clay loam	35	20	15	36	22	4428	2706	

FC, field capacity; PWP, permanent wilting point; TAW, total available water. *Soil water thresholds adapted from: Ratliff et al. (1983) and Hanson et al. (2000).

### Hazelnut Phenology

To record the phenological phases of *C. avellana*, the authors used a modified version of the phenological guide of Puppi and Zanotti (1998). For this study, the development of the female flowers was classified into the following stages: Stage R7, beginning of female flowering (the red tips of the stigmas are visible); Stage R8, Inflorescence in full bloom (stigmas are fully extended); Stage R9, end of the flowering (stigmas are wilted and dark red); Stage R10, ovaries enlarge (the inflorescence start to evolve into clusters); Stage R11, small cluster are visible; Stage R12, immature fruits (fruits are visible but still green); Stage R13, fruits reach maturity (the shell turn brown and harden). The phenology was recorded every week.

### Pollen

**FIGURE 1:**
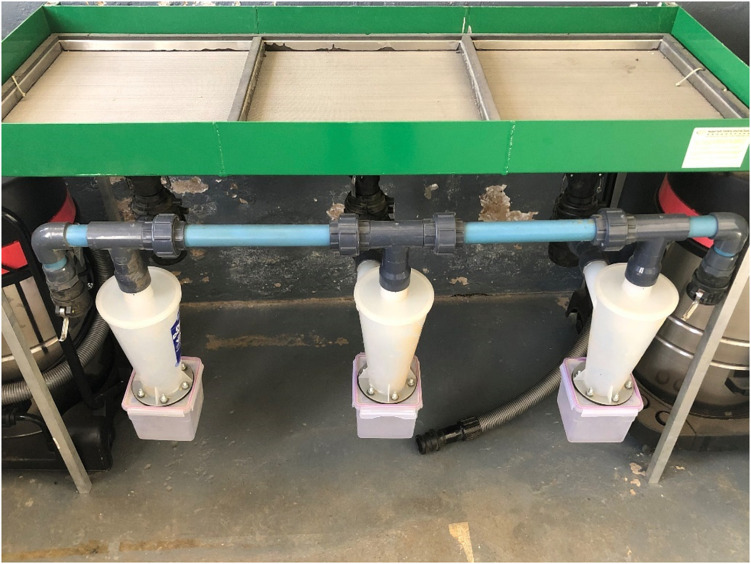
Machine built to extract hazelnut pollen.

Pollen was collected during winter time from Barcelona (S_1_ S_2_), Hall’s giant (S_5_ S_15_), Nocchione (S_1_ S_2_), and Tonda Gentile Romana (S_10_ S_20_). Catkins were dried at room temperature overnight. To optimize the pollen collection, a machine was locally developed (Figure 1), consisting in a series of filters connected to a hopper (100 and 50 μm). The size of pollen grains of *Corylus* spp. can vary in size from 20–25 × 26–28 μm (Kubik-Komar et al., 2018). At the bottom, a Dust Deputy DIY Cyclone Separator was attached. At the top end of the dust separator (Oneida Air System, United States), a flexible anti-static pipe was connected with a wet/dry vacuum machine (>1800 W). At the lower end of the cyclone dust separator, 1-L plastic container was attached to it, where thanks to the centrifugal force, the pollen ends. Before starting the process, the catkins were shredded using a shredding mulching box (Ryobi RGS-1240, 2400 W). After extraction, the pollen was vacuum packed and stored at −20°C (Novara et al., 2017).

### Pollen Suspension and Assisted Pollination

For the suspension, the procedure followed the method described by Ascari et al. (2018). The pollen was suspended at 0.1% (w/v) in a liquid media containing: 10% (w/v) sucrose mixed with 0.5% (w/v) xanthan gum and 0.02% boric acid. The applications were carried out when the female inflorescences were in full bloom (Phenological stage: R8). For Tonda di Giffoni, the treatments started mid-June and for Barcelona cultivar mid-July. The application was done during late afternoon, when environmental humidity and temperature were more favorable. The pollen suspension was applied using mist blowers (STIHL, SR420). Fifty liters of solution per hectare were required to complete the application. The experiment was carried out in an area without natural pollinators. Based on the amount of pollen available, for each test, 150 g pollen ha^–1^ was applied.

### Observation of Pollen Tube Growth

After the assisted pollination, female inflorescences were sampled at 48 h, 5 days, 10 days, and 15 days. In the orchard, 10 plants for each block and treatment were randomly selected. From each tree, 10 flowers were collected from 1-year old branches. To control the border effect, the border rows and the ones between two net sections were not sampled. Samples were stored in a portable cool box and taken to the laboratory. The following procedure followed the method of Liu et al. (2012, 2014). The inflorescences were fixed in FAA solution for 3 days, and then stored in 70% alcohol at 4°C. After 24 h, the samples were rehydrated in 50-30-15% ethanol, for 20 min each step. The inflorescences were then observed under a stereo microscope (LEICA, EZ4) and with the aid of a scalpel, the pistils were separated from the inflorescences (Figure 6C). The pistils were socked in Eppendorf tubes containing 5 M NaOH solution and kept at 35°C for 3 days (Figures 6D–F). The samples were rinsed three times in distilled water and macerated for 30 s in 0.1 M acetic acid solution. Subsequently, the pistils were further washed in distilled water three times and stained in aniline blue solution (0.1 g aniline blue + 0.071 g K_3_PO_4_ + 100 mL distilled water) for 24 h. Finally, the samples were observed with an OMFL600 Inverted Fluorescence Compound Microscope with a set of UV filter, and equipped with a digital microscope camera (Summit SK2-10 × 10.0MP PC) (Figures 6G–J).

### Observation of Disodium Fluorescein During Nut Development

Once the fruit clusters were visible, samples were taken following the sampling pattern mentioned for the inflorescences. Unlike the female flowers, clusters were collected with a piece of stalks, ±5 cm long. Immediately, samples were placed in jars containing water to prevent embolism and taken to the laboratory. Subsequently, the end of the stalks was inserted in 50 mL glass jars containing a solution of 0.25% of disodium fluorescein and placed in a growing tent at 25°C and 60% RH. The tent was illuminated for 24 h, using full spectrum grow lights (MarsHydro reflectors, 300 W^1^). Finally, the fruits were longitudinally dissected and observed under the inverted fluorescence compound microscope.

### Statistical Analysis

For the longevity of the stigma’s receptivity among the treatments, a survival analysis was performed using GraphPad Prism version 8.4.3 for Windows (GraphPad Software, San Diego, CA, United States). Log-rank test was adopted to compare the distribution of the samples. Regarding the observations of the pollen tube growth and nut development, statistical significance of the effect of colored shade nets, ANOVA analysis was carried out using XLSTAT (Addinsoft, New York, NY, United States). Tukey’s HSD test was applied to all pairwise differences between means. Dunnett’s test was performed to compare each category with the control category. Means were compared using least significant difference (LSD).

## Results

### Temperature and Relative Humidity Under Photo-Selective Nets

**FIGURE 2:**
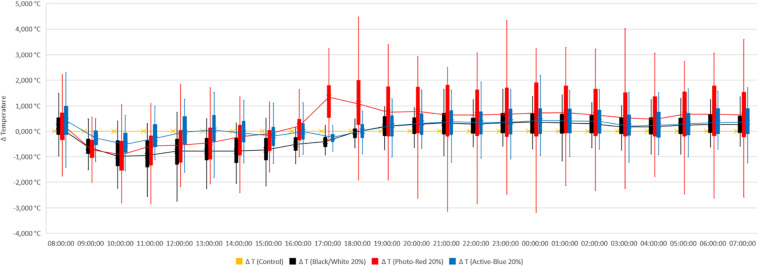
Temperature recorded under photo-selective nets during winter (from June to end of August). Data were collected hourly. Temperature is expressed as difference between temperatures recorded under the nets and in open field (control). The horizontal middle line represents the median that goes through the mean of each box. The whiskers (vertical lines) extend from the ends of the box to the minimum value and maximum value.

**FIGURE 3:**
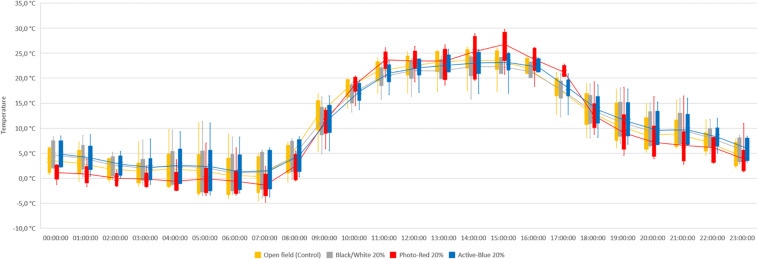
Temperature recorded under photo-selective nets during a warm spell in winter. Data were collected hourly for a week. The horizontal middle line represents the median that goes through the mean of each box. The whiskers (vertical lines) extend from the ends of the box to the minimum value and maximum value.

**FIGURE 4:**
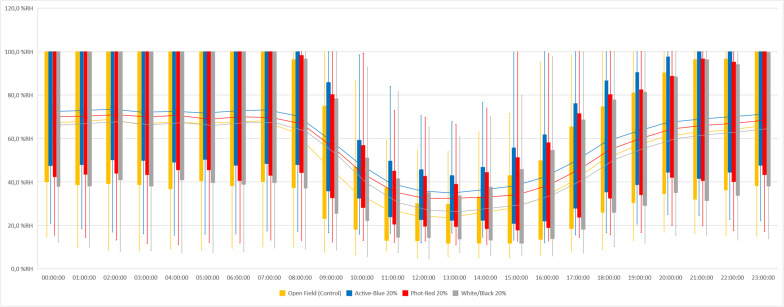
Relative humidity measured under photo-selective nets and in open field (control) in winter (from June to end of August).

In this study, differences in temperatures up to 6°C were observed between the nets and the open field (control) (Figure 2). Variations also occurred between the types of photo-selective nets (Table 3). During winter (from June to end of August), the average temperature recorded between 8 am and 4 pm was higher in the open field (T_Max_: 28.6°C; T_AVG_: 17.3°C; T_Min_: −3.4°C), lower under the Active-Blue net (T_Max_: 27.9°C; T_*AVG*_: 17.1°C; T_Min_: −2.8°C), moderately lower under the Photo-Red net (T_Max_: 29.8°C; T_*AVG*_: 16.8°C; T_Min_: −2.2°C), lower under the White/Black net (T_Max_: 27.4°C; T_AVG_: 16.5°C; T_Min_: −3.1°C). During night hours, the average temperatures recorded between 6 pm and 7 am were higher under the red net (T_*Max*_: 22.2°C; T_*AVG*_: 7.8°C; T_Min_: −4.8°C), moderately lower under the blue net (T_*Max*_: 2.1°C; T_AVG_: 7.4; T_Min_: −5.5°C), lower under the white/black net (T_*Max*_: 22.3°C; T_AVG_: 7.3°C; T_Min_: −5.7°C), and slightly lower in open field (T_*Max*_: 22.3°C; T_AVG_: 7°C; T_Min_: −5.6°C). Interesting was to observe a temperature increase induced by the red net between 4pm and 5pm, followed by a progressive decrease. To summarize, during daytime, the nets were able to slightly reduce the temperature compared to the open field. However, in the presence of particular heat waves in winter (Figure 3), the daily mean temperatures under the Phot-Red were slightly higher compared to both, the control and the other nets (Figure 3). When these events were occurring, the fluctuation between day and night under the red net was accentuate, recording lower temperatures compared to the other tests. RH was recorded simultaneously to temperature. Sensors revealed that RH% changed cyclically during the day, increasing during the night hours while decreasing during the sunlight exposed hours (Figure 4). In average, under the netted areas, an increase of daily relative air humidity by approximately 2–10% was recorded. From 8 am, RH decreased sharply reaching the minimum (<10%) at 3–4 pm. During sunset, RH started to increase again. Among all the tests, the RH means were constantly higher under blue net (Figure 4).

**TABLE 3:** Results of ANOVA for the effect of treatment setup on temperature differences and relative humidity.

Treatments comparison	Temperature (significance level)	Relative humidity (significance level)
Control vs. Black/White 20%	<0.0001	0.9469
Control vs. Photo-Red 20%	<0.0001	<0.0001
Control vs. Active-Blue 20%	0.9024	<0.0001
Black/White 20% vs. Photo-Red 20%	<0.0001	0.0010
Black/White 20% vs. Active-Blue 20%	<0.0001	<0.0001
Photo-Red 20% vs. Active-Blue 20%	0.0003	0.0181

### Stigmas Receptivity

**FIGURE 5:**
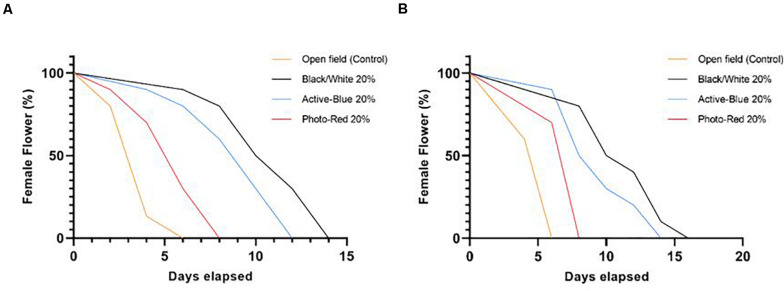
Stigmas receptivity among the different treatments. Data were collected during phenological recording. The days were counted starting from the phenological stage R8 (full bloom), until stage R9 (stigmas are wilted and dark red). **(A)** cv. Tonda di Giffoni; **(B)** cv. Barcelona. Log-rank (Mantel-Cox) test, *P* < 0.0001.

**FIGURE 6:**
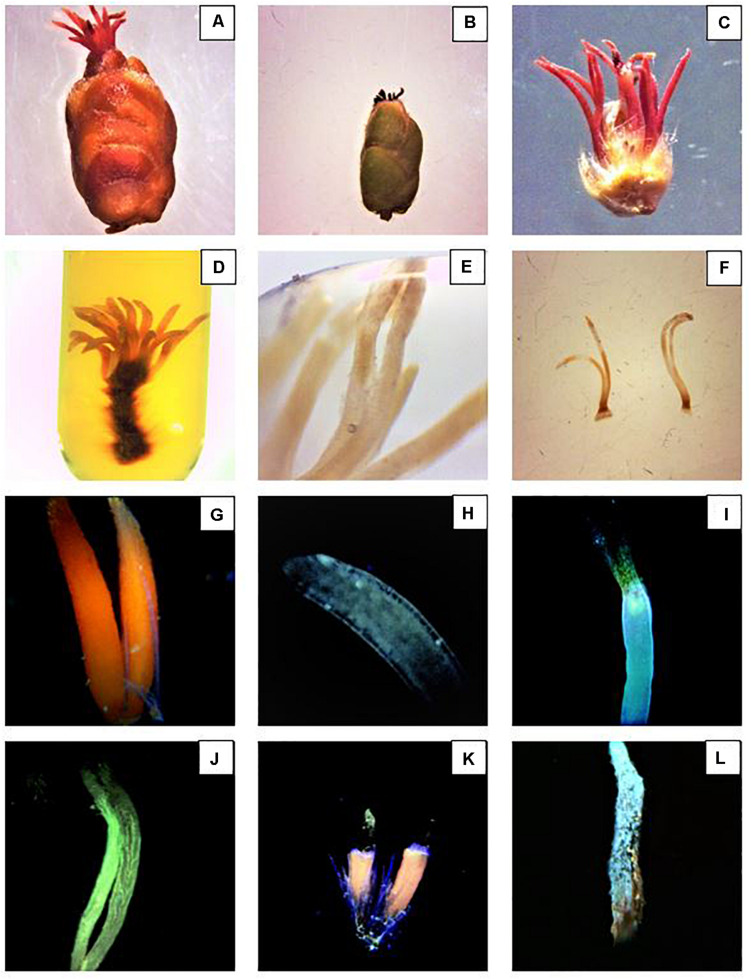
Preparation and examination of the stigmatic styles. **(A)** Hazelnut female inflorescence in R8 stage. **(B)** Female inflorescence after dust application. **(C)** Structure of the female inflorescence after bud dissection. **(D)** Stigmatic styles soaked in 5 M NaOH solution after 2 days. **(E)** Stigmatic styles soaked in 5 M NaOH solution after 3 days. The bleaching phase is a fundamental step to properly visualize the pollen tubes. **(F)** Samples ready to be squashed in aniline blue. **(G,H)** Healthy non-pollinated stigmatic styles. **(I)** Top of the style withered and the pollen tube curled and arrested at the top part of the style. **(J)** Pollen tubes reached the base of the styles. **(K)** Damaged stigmatic styles covered with dust. **(L)** Pollen grains siting on a dried style after dust application.

During winter 2018 and 2019, phenological data reported that the full bloom (Stage R8) was observed in mid-July for Tonda di Giffoni and at the beginning of August for Barcelona. At this stage, the female flowers showed the typical red stigmatic styles poking out (Figures 6A,C) from the bud scales (Olsen, 2013). In areas where hazelnut is traditionally cultivated, the female flowers can remain receptive for few months (Thompson, 1979; Hampson et al., 1993). In this study, due to the warmer and dry winter condition during daytime, the stylar desiccation was accelerated. In fact, in open field, the female flowers maintained their red appearance for no longer than 1 week (Figure 5). In some cases, the tips of the styles turned black during the stage R7. Under the netted blocks, the reduced solar irradiation, the slightly cooler, and higher humid conditions prevented such rapid stylar desiccation, extending the flowers receptivity up to 2 weeks. Obviously, these differences affected the capacity of the styles to offer a good support for pollen grain adhesion and germination and showed how slight changes in temperature and humidity modifications can influence the success of pollination in hazelnut.

### Observation of the Pollen Tube Growth

The observations were made at 48 h, 5 days, 10 days, and 15 days after the pollen application. For each step, 50 female inflorescences were collected for each cultivar and treatment. Each experiment had four replicas. In total, 200 glomerules were examined for each step. At 48 h, significant differences in pollen germination rate were recorded between open field and netted areas (Tables 4, 5). Generally, germination was most affected in open field; on the contrary, the highest values were recorded under Black/White net (*p* < 0.0001). The Black/White net performed better than the Photo-red net for both cultivars (TdG: *p* < 0.001; Bar: *p* < 0.0001). In Tonda di Giffoni, Active-blue net showed detectable variation compared to the open field condition (*p* < 0.001); however, no statistically significant differences were detected in comparison with the other two nets. On other hands, Barcelona under the blue net showed higher pollen germination levels (*p* < 0.001), where no differences were found between the red net and the control. After 5 days, the number of samples showing pollen tubes reaching the base of the styles was generally low. However, the higher amount was recorded under the Black/White net (*p* < 0.0001). Analogous results were found for the blue net. For both cultivars, the red net did not show significant results compared to the control under direct sunlight. In the 10-days check, Tonda di Giffoni and Barcelona showed the same trend. Black/White net gave the higher number of pollen tubes reaching the base of the styles (*p* < 0.0001), followed by the blue net (*p* < 0.001) and the red net (*p* < 0.025). The samples collected after 15 days did not show a substantial increment in pollen tubes reaching the base of the styles compared to the 10 days check point.

**TABLE 4:** Pollen germination mean after 48 h.

Treatment	Tonda di Giffoni	Barcelona
Black/White 20%	135.500a***	159.500a***
Active-Blue 20%	120.500ab**	143.250b***
Photo-Red 20%	107.250b*	119.250c
Open field (Control)	90.250c	117.500c
Pr > F(Model)	***p < 0,0001; **p < 0.001; *p < 0.05.	

*Summary LS means [Tukey (HSD)]. The letters show statistically significant differences between variables.*

**TABLE 5:** Pollen tubes that reached the base of the styles at 5, 10, and 15 days after pollination.

	5 days		10 days		15 days	
			
Treatment	Tonda di Giffoni	Barcelona	Tonda di Giffoni	Barcelona	Tonda di Giffoni	Barcelona
Black/White 20%	51.75a***	72.96a***	144.5a***	203.7a***	152.25a***	211.45a***
Active-Blue 20%	46.25a***	65.2a***	133.25ab**	188.38ab**	138.75ab**	193.88ab**
Photo-Red 20%	32b	45.12b	120b*	166.7b*	123.5b*	170.2b*
Open field (Control)	23.75b	33.48b	99c	139.59c	102c	142.59c
Pr > F(Model)	***p < 0,0001; **p < 0.001; *p < 0.05.					

*Summary LS means [Tukey (HSD)]. The letters show statistically significant differences between variables.*

### Observation of Disodium Fluorescein During Nut Development

**FIGURE 7:**
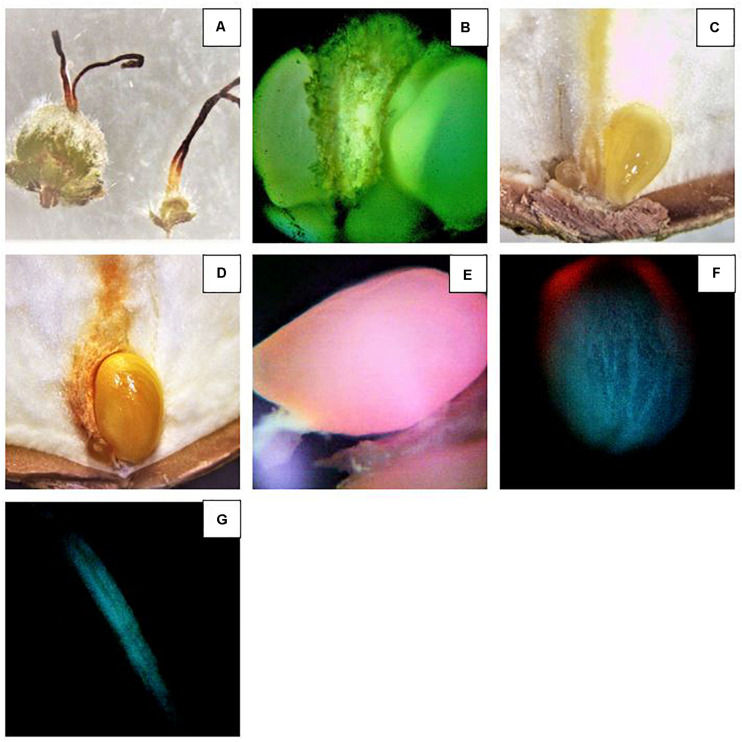
Investigation of the ovule development using disodium fluorescein. **(A)** Comparison between an abortive ovary (right) and developing ovary (left). **(B)** Intense fluorescence observed in two developing ovules. **(C)** Two ovules. The one on the right is developing and the other on the left side, much dimmer, ceasing the development. Note the yellowish color of the funicle and developing ovary after disodium fluorescein absorption. **(D)** Two ovules. The one on the right continue to develop and the other on the left side ceased the development. **(E–G)** Reduced transport of disodium fluorescein in immature ovules ceasing the development **(E,F)** and a funicle where the ovule completely ceased the development.

To study the nut development, disodium fluorescein was utilized as indicator of vascular continuity as described by Liu et al. (2013). The advantage of this technique is that in 24 h, disodium fluorescein is transported in functional vascular tissues and it is easily detected in tissues at very low concentration thanks to the strong fluorescence under the microscope (Figure 7). As result, the presence or absence of fluorescence is reliable indicator that transport is occurring or not (Figure 7). Strong fluorescence could be observed in the viable ovules. At R11, clusters became visible and the two ovules could be easily observed thanks to the bright fluorescence (Figure 7B). Interestingly, no differences were recorded between the treatments. The only detail worthy of note is that almost all the samples showed one ovule smaller than the other. Significant differences were observed at R12 (Table 6). A markedly difference between the diameters of the two ovules in the same ovary could be easily observed (Figures 7C,D). The fluorescence intensity differs between the two. In fact, the fluorescence in the smaller ovule was slightly dimmer. Moreover, blank nuts could be easily detected. In this case, the developing nuts showed a very dim fluorescence or nothing (Figures 7F,G). In both cultivars, Tonda di Giffoni and Barcelona, the highest number of fruits showing continuity of transport was recorded under the Black/White net (*p* < 0.0001) and Active-blue net (*p* < 0.0001). No significant differences were observed between the two nets. Considerable differences were observed between the Photo-red net and the open field (*p* > 0.043). However, statistically significant differences were recorded between the Black/White or Active-blue net and the Photo-red net (*p* < 0.0001), which performed poorly compared to the other two types of nets.

**TABLE 6:** Mean of disodium fluorescein detected in viable ovule during the phases R11 and R12.

	R11		R12	
		
Treatment	Tonda di Giffoni	Barcelona	Tonda di Giffoni	Barcelona
Black/White 20%	184.5a	190.98a	105.965a***	123.304a***
Active-Blue 20%	181.5a	187.39a	100.663a***	116.075a***
Photo-Red 20%	174.0a	179.64a	63.430b*	73.803b*
Open field (Control)	171.0a	177.01a	47.613c	55.038c
Pr > F(Model)	***p < 0.0001; **p < 0.001; *p < 0.05.			

*Summary LS means [Tukey (HSD)]. The letters show statistically significant differences between variables.*

## Discussion

Contrary to more common hail nets, photo-selective nets are usually deployed to decrease light intensity and to alter the spectrum, to buffer extreme temperatures and increase RH (Shahak et al., 2004, 2008; Elad et al., 2007; Wachsmann et al., 2014; Ilić et al., 2015). In warm temperate production regions of South Africa, climate warming is affecting accumulation of chill units and RH, eventually reaching a critical threshold for temperate deciduous fruit and nut trees (Midgley and Lötze, 2011; Midgley et al., 2016). The pollination mechanism can be critically affected under high air temperature, reducing stigmatic receptivity and pollen germination (Pham et al., 2015; Mditshwa et al., 2019). Elevated temperatures often take place in combination with high solar radiation, drought, and strong wind, which can be harmful for the plant and especially for the pollination process (Hall, 1992; Devasirvatham et al., 2012; Monteiro et al., 2015). Previous research demonstrated how *C. avellana* can drop more than 80% of the flowers, even if they show a developed pollen tubes in the styles (Rimoldi, 1921; Romisondo, 1965; Thompson, 1967) and how the percentage of the blanks produced can be influenced by abiotic stress and resources availability (Painter, 1956; Silva et al., 1996; Erdogan and Mehlenbacher, 2001; Beyhan and Marangoz, 2007; Liu et al., 2012, 2014). Mild temperature and high humidity are required for the pollen tube to grow quickly and complete successfully the process (Huo et al., 2014). Generally, the pollen germinates in 1–2 days (Kelley, 1979) and it takes from 2 to 10 days for the pollen tube to reach the base of the style (Figure 6J) (Rigola et al., 1998; Liu et al., 2014). Çetinbaş-Genç et al. (2019) demonstrated that pollen tubes in hazelnut are sensitive to high temperature and how the pollen tolerance varies between cultivars. Huo et al. (2014) proved the influence of RH and temperature on the stigma receptivity and pollen tube growth in *Corylus kweichowensis* Hu. Basically, the health and the speed at which the pollen germinates and the pollen tube grow depends on weather conditions during winter, which can considerably affect the fate of reproduction and fertilization processes (Boyer, 1982; Hopf et al., 1992; Silva et al., 1996; Delph et al., 1997; Giorno et al., 2013; Rieu et al., 2017). In this study, photo-selective technology was tested for the first time in a hazelnut orchard with the aim to modify microclimatic conditions and explore the netting effect on the pollination process. Artificial pollination was carried out utilizing a suspension media. This method showed to be more effective than dry-pollen applications, especially when warm and dry winter conditions threaten the pollen viability (Ascari et al., 2018). In accordance with other authors, the augmented stigma receptivity, pollen germination, tube growth, and nut development under some of the nets could be probably associated to the decreased air/canopy temperature and increased RH (Germanà et al., 2003; Zilkah et al., 2013). Under black/white net slightly lower T_*Max*_ and T_*AVG*_ were recorded. Moreover, the mean temperatures were generally lower between 10 am and 4 pm, the hottest time of the day (Figure 2). In addition, black/white net showed an improved flower receptivity, pollen germination, pollen tube development, and detected viable ovules (Tables 4–6). Active-blue net performed slightly lower compared to the black/white net in terms of the reproductive parameters even though the RH% recorded was generally higher. Instead, Photo-red net was generally exhibiting mean temperatures lower than open field conditions during the daytime, showing an unusual pick between 16:00 and 19:00. T_*Max*_ was higher under red net compared to the other two treatments. In case of warm spells in winter, the red net showed temperature even higher than open field conditions. Moreover, the receptivity of the female flowers, pollen germination, tube development, and viable ovules under red nets were closer to under sunlight conditions. This variability under the nets may be correlated to the net color and its spectral quality (McDonald, 2003). The effect of Photo-Red net could alter the flowering period (Smith and Whitelam, 1997; McDonald, 2003; Mupambi et al., 2018; Kalaitzoglou et al., 2019). For this reason, further studies are necessary to investigate the effect of net color and spectral quality on the reproductive biology of hazelnut. Nevertheless, the performance of photo-selective nets and their influence on certain physiological parameters of a tree seem to vary based on the geographical location (Meena et al., 2015; Zoratti et al., 2015; Brkljača et al., 2016). For this reason, in the absence of any existing local information, it is necessary to verify the effects of photo-selective nets on the targeted crop. As mentioned above, RH is another important parameter affecting the reproductive biology of *Corylus* spp. Although the nets had analogous shade factor, variation in RH% was detected (Table 3). Hazelnut trees grown under Active-blue nets had a modest higher RH%, followed by the red and the black/white nets. Nevertheless, these differences in RH% did not give any significant improvement in terms of reproductive parameters. Previous studies carried out by Middleton and Mcwaters (2002) and Rigden (2008), in comparison to open field conditions, showed how shade nets increased RH by 10–15%. However, in this study when a warm spell was occurring during winter, the effect of the nets was drastically reduced. A remarkable factor was the reduced water use under the netted area. During the dry winter season, when trees are dormant, because of the reduced solar radiation reaching the orchard floor in combination with a reduced wind speed, the irrigation input was greatly reduced (Table 2). Another factor that could affect the longevity of the female flowers and interfere with the pollination process, especially in field condition, was the presence of dust (Waser et al., 2017; Zhang et al., 2019). Hazelnut is a wind pollinated plant and the sticky style exposed to air can be polluted by dust. Observing the styles under the microscope (Figures 6K,L), large amount of dust particles was detected. Further studies are necessary to investigate how it affects hazelnut pollination. In conclusion, the results of this study bring out that the use of photo-selective nets could represent an interesting practice to assist the plants during sensitive phases, such as flowering and pollination. However, the results should be considered preliminary and verified by further studies in the coming years. Much more need to be explored, especially focusing on the effect of light variations by the nets on flowering, alternate bearing, risk of cluster abscission, final yield, quality, and nutritional value of the nuts.

## Data Availability Statement

The raw data supporting the conclusions of this article will be made available by the authors, without undue reservation.

## Author Contributions

DG was the principal investigator. DG, JA, and GE contributed to conception and design of the study. GE and OS contributed to the design of the net structures. ES contributed and organized the pollen collection. MS and OS organized the data collection and organized the database. DG performed the statistical analysis and wrote sections of the manuscript. ES contributed to writing the introduction of the manuscript. All authors contributed to manuscript revision, read, and approved the submitted version.

## Conflict of Interest

DG is employed by Ferrero Hazelnuts Company, Division of Ferrero Trading Luxembourg and Agrisudafrica Ltd. ES and JA are employed by Ferrero Hazelnuts Company, Division of Ferrero Trading Luxembourg. MS, OS, and GE are employed by Agrisudafrica Ltd.

## Footnotes

1 www.mars-hydro.com
